# Development of a Compartmental Model to Investigate the Influence of Inflammation on Predictions of Vitamin A Total Body Stores by Retinol Isotope Dilution in Theoretical Humans

**DOI:** 10.1093/jn/nxaa407

**Published:** 2021-01-20

**Authors:** Michael H Green, Jennifer Lynn Ford, Joanne Balmer Green

**Affiliations:** Department of Nutritional Sciences, College of Health and Human Development, The Pennsylvania State University, University Park, PA, USA; Department of Nutritional Sciences, College of Health and Human Development, The Pennsylvania State University, University Park, PA, USA; Department of Nutritional Sciences, College of Health and Human Development, The Pennsylvania State University, University Park, PA, USA

**Keywords:** humans, inflammation, retinol-binding protein, vitamin A status, WinSAAM

## Abstract

**Background:**

Inflammation, both acute and chronic, is associated with reductions in the synthesis of retinol-binding protein (RBP) and the concentration of retinol in plasma. Consequently, it is currently recommended that the retinol isotope dilution (RID) method not be used to estimate vitamin A total body stores (TBS) in subjects with inflammation.

**Objectives:**

To apply compartmental analysis to study the effects of inflammation on hepatic apo-RBP input, plasma retinol pool size, and RID-predicted TBS in theoretical subjects with known steady state values for these parameters.

**Methods:**

We selected 6 previously generated hypothetical subjects who ingested a dose of stable isotope–labeled vitamin A (day 0). Starting with each subject's published steady state model for retinol tracer kinetics, we developed a parallel model for unlabeled retinol and RBP that incorporated links between these entities and tied liver retinol secretion to RBP availability. Then we perturbed the steady state model by initiating chronic or acute inflammation on day 0 or acute inflammation on day 3 or 9 and simulating results for RBP, plasma retinol, and TBS.

**Results:**

Chronic inflammation led to substantial reductions in RID-predicted TBS for at least 2 weeks after tracer administration. In contrast, acute inflammation induced on day 0 or 3 resulted in less dramatic impacts on TBS (37% or 20% reduction, respectively, from steady state levels, with levels rebounding by 14 days). When inflammation was induced 9 days after administration of the tracer, the effects on predicted TBS were minimal. Overall, for acute inflammation initiated at 0, 3, or 9 days, accurate predictions of TBS were obtained by 2 weeks.

**Conclusions:**

Compartmental analysis can be applied in the novel way described here to study the influence of perturbations such as inflammation on predictions of vitamin A status using RID. Such an approach has potential value for studying other perturbations and different nutrients.

## Introduction

It is well known that inflammation affects vitamin A metabolism (1) and that inflammatory conditions are often present in populations whose vitamin A status is of interest. Because of the influence of inflammation on plasma retinol and retinol-binding protein (RBP) concentrations, it has been suggested (2, 3) that some methods used to assess vitamin A status [including retinol isotope dilution (RID)] may not provide accurate results when inflammation or infection is present.

Since RID is currently accepted as the best available and most feasibly applied method for estimating vitamin A total body stores (TBS) in humans (3, 4), a better understanding of the impact of inflammation on RID-predicted TBS is vital. The RID technique, which was originally proposed by Bausch and Rietz (5), developed by Furr et al. (6), and subsequently refined by others (7–10), involves measurement of plasma retinol specific activity (SA_p_) at a designated time after subjects ingest stable isotope–labeled vitamin A; then SA_p_ is used in a TBS prediction equation that includes several factors (coefficients) related to vitamin A absorption and retention and to the ratio of retinol specific activity in plasma versus stores.

In a previous study (10), the RID equation shown here as Equation 1 was used to estimate vitamin A stores in young adults:

(1) $$\begin{eqnarray*}
{\rm{TBS}}\,\left( {{\rm{\mu mol}}} \right){\rm{ }} = FaS\, \times \,{\rm{1/S}}{{\rm{A}}_{\rm{p}}}
\end{eqnarray*}$$

Here, *Fa* is the fraction of the isotope dose present in stores at the time of blood sampling and *S* is the ratio of retinol specific activity (fraction of dose/μmol) in plasma (SA_p_) to that in stores (SA_s_) at the same time; it was recommended that SA_p_ be measured 4 or 5 days after dosing. When RID is done in community settings [e.g., see Van Stuijvenberg et al. (11) and references therein], 1 blood sample is obtained from each subject for the measurement of SA_p_ and values for the RID coefficients are derived from the literature (6). In contrast, Green et al. (10) calculated values for *Fa* and *S* using model-based compartmental analysis (12); the coefficients can also be obtained by applying population modeling (“super-subject” approach) to group mean data (13).

Previous research, reviewed in Rubin et al. (1), indicates that inflammation, which can be assessed in field studies by measuring biomarkers such as C-reactive protein (CRP) and a1-acid glycoprotein (AGP), may influence absorption of both dietary and labeled vitamin A and that inflammation leads to a transient (acute inflammation) (14, 15) or more prolonged decrease (chronic inflammation) in plasma retinol concentration; the latter is linked to a decrease in the production of RBP, a negative acute-phase protein, resulting in lowered secretion of holo-RBP by the liver (14, 16).

In light of concerns that inflammation might compromise the accuracy of RID predictions of TBS, we sought to advance our previously published compartmental models for vitamin A kinetics (13, 17, 18) by incorporating simulations reflecting responses to inflammation, specifically focusing on the influence of inflammation on RBP production and RID-predicted TBS. To accomplish this, we applied model-based compartmental analysis to data for several previously studied theoretical subjects (18, 19) and simulated the generalized impacts of chronic and acute inflammation—as well as the timing of administration of the tracer dose versus the onset of inflammation—on the kinetics of plasma retinol (tracer and tracee), on hepatic RBP input, and on the estimates of vitamin A status obtained by RID. In previous work (19–21), we have shown that by analyzing simulated data for hypothetical subjects, we can confirm the accuracy of the RID method for estimating TBS; we were thus confident that this approach would provide useful information related to generalized examples of inflammation. In addition to those results, we present, for the first time insofar as we know, a compartmental model that links hepatic RBP input to the secretion of diet-derived retinol from hepatocytes and the recycling of stored vitamin A from hepatic stellate cells. This work lays the groundwork for studying the influence of other perturbations on the vitamin A system.

## Methods

### Theoretical data

**FIGURE 1:**
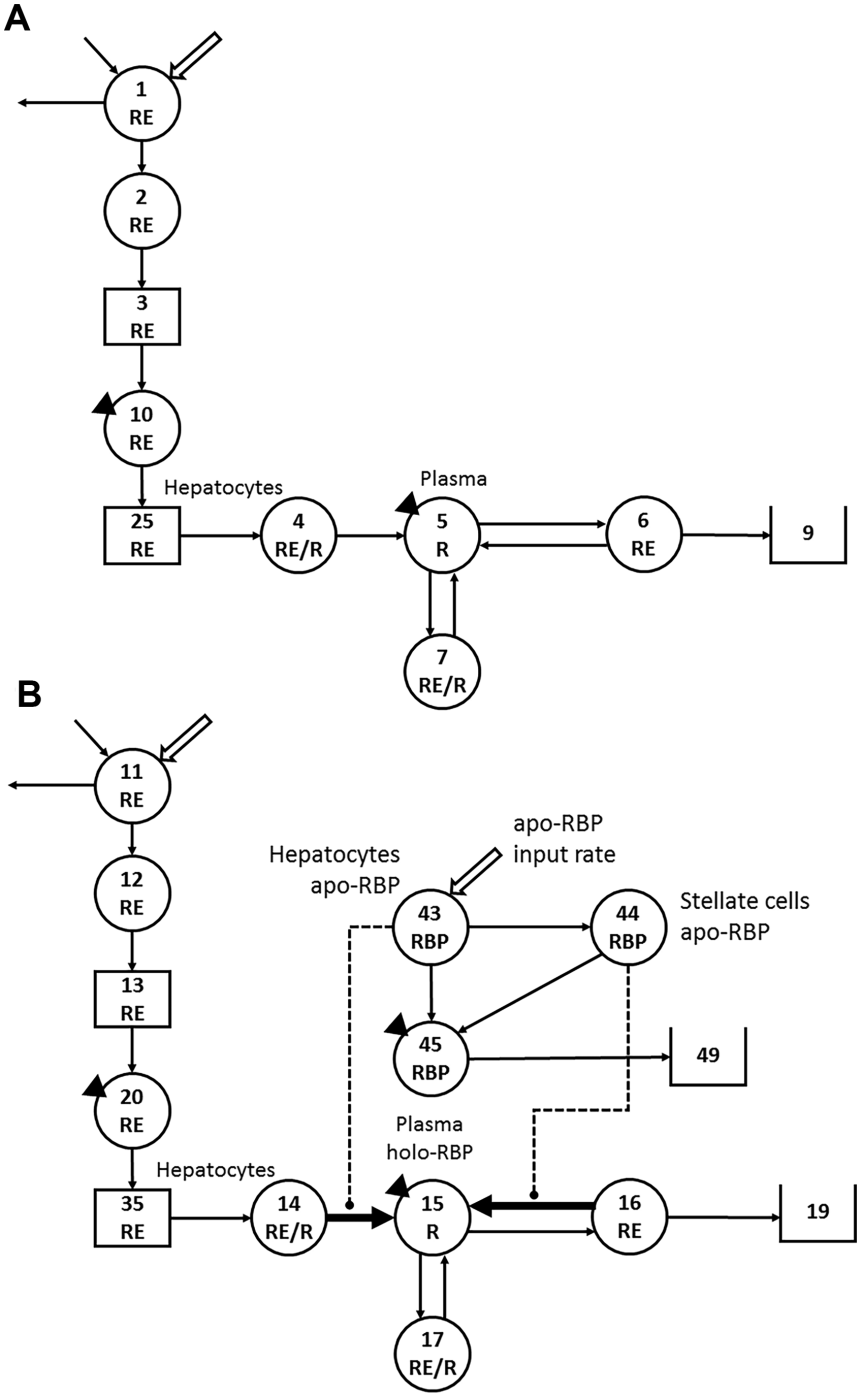
Working compartmental models for whole-body vitamin A metabolism (A) developed from retinol tracer data versus time for theoretical human subjects and (B) developed for unlabeled retinol and RBP based on the retinol tracer model. (A) Model adapted from Green et al. (18). Circles represent compartments; the rectangles are delay elements; interconnectivities between components (arrows) are fractional transfer coefficients [L(I,J)s, or the fraction of retinol in compartment J transferred to compartment L each day]; and DT(I)s are delay times (or days spent in delay element I). (A) Components 1–3 represent the processes of vitamin A digestion and absorption plus the production of chylomicrons; compartment 1 is the site of introduction of the vitamin A dose and dietary vitamin A, as well as the site of loss for unabsorbed vitamin A. Chylomicrons are metabolized in plasma component 10 before hepatocyte uptake of RE in chylomicron remnants (delay element 25 and compartment 4). Retinol bound to RBP is secreted from compartment 4 into plasma retinol compartment 5; R in plasma can exchange with vitamin A in 2 extravascular pools (a larger compartment 6 and a smaller compartment 7); irreversible loss from the system is from compartment 6 into sink 9. The triangles indicate that plasma is the site of sampling (compartment 10 for RE and compartment 5 for R). (B) A parallel model for unlabeled retinol (tracee; compartments 11, 12, 20, 14, 15, 16, and 17; components 13 and 35; and sink 19), with compartments for RBP added. For unlabeled retinol, interconnectivities represent transfer rates (μmol retinol or RBP/d). For apo-RBP, input (synthesis) is into hepatocyte compartment 43. From compartment 43, RBP can pick up retinol from compartment 14 for secretion into plasma compartment 45 at the same rate as retinol enters plasma compartment 15 from hepatocyte compartment 14, or apo-RBP can be transferred to hepatic stellate cells (compartment 44), where it can acquire retinol from stores (compartment 16) and enter plasma compartment 45 as holo-RBP, paralleling the rate of transfer of retinol from compartment 16 into compartment 15. The thick arrows indicate L(I,J)s that could be time-variant depending on the condition; the dashed lines indicate control elements that link the RBP and retinol models and are regulated by the size of the hepatocyte and stellate cell apo-RBP pools. Abbreviations: DT, delay time; R, retinol; RBP, retinol-binding protein; RE, retinyl esters.

We selected 6 previously studied healthy theoretical subjects (18, 19) who had been assigned normal values for plasma retinol concentrations, a range of ages (subjects 4–6) (19) or vitamin A absorption efficiency (subjects 1–3) (18), low to high values for TBS, and varied retinol kinetics and state variables (**Supplemental Table 1**). In the original studies, WinSAAM [the Windows version of the Simulation, Analysis and Modeling software; www.winsaam.org (22–24)] was used to simulate data for plasma retinol tracer kinetics versus time after subjects ingested a dose of stable isotope–labeled retinyl acetate; data were simulated in light of a 9- (18) or 7-component model (19) for whole-body vitamin A metabolism and included a steady state solution so that TBS predicted by the model or calculated using an RID equation with the correct time-variant coefficients would be constant over time. For the current project, data for subjects from (19) were resimulated in light of the 9-component model shown in **Figure 1**A (tracer model) and data for all subjects were plotted over 30 days. As described in detail in the figure legend, the retinol tracer model includes 7 compartments (compartments 1, 2, 4–7, and 10), 2 delay components (components 3 and 25), and a loss sink (component 9); compartment 1 is the site of input of tracer; interconnectivities between components are described by fractional transfer coefficients [L(I,J)s, or the fraction of retinol in compartment J transferred to compartment I each day]; and delay times [DT(I)s] are the time (in days) the tracer spent in delay element I. Using the assigned values (Supplemental Table 1) for plasma retinol pool size as input for a steady state solution in WinSAAM, we obtained values for sizes of other vitamin A pools [M(I)s; μmol], including TBS, and for rates of vitamin A transfer between compartments [R(I,J)s; μmol/d].

### Development of models for unlabeled retinol and for RBP

In light of the fact that RBP is required for retinol secretion from the liver and because plasma concentrations of both RBP and retinol are reduced during inflammation, we set out to further advance our compartmental model for vitamin A metabolism to include liver RBP input and secretion. Since we did not have published data for RBP, we began by first using the retinol tracer model and the data for subject 2 to develop a parallel model (Figure 1B) for unlabeled retinol (tracee), similar to the approach used in (21, 25). The parallel tracee model includes compartments 11, 12, 14–17, and 20; delay components 13 and 35; and loss sink 19, with dietary vitamin A input into compartment 11. We set tracee model parameters equal to those for the corresponding values from the tracer model and included model-predicted steady state pool sizes as initial conditions in each compartment and delay element.

We next embedded compartments representing RBP into the retinol tracee model, incorporating the currently accepted knowledge that apo-RBP is required for and can be rate limiting for secretion of retinol from hepatocytes and liver stellate cells. The model also accommodated information in the literature related to effects of inflammation on hepatic RBP production (15) and on plasma RBP and retinol concentrations (26). In Figure 1B, RBP input into the system [U(43)] is shown into hepatocyte compartment 43. The basal rate of apo-RBP input and distribution between hepatocytes and stellate cells was based on the model-predicted rate of retinol input into plasma from diet [R(15,14)] plus the rate of retinol recycling from the larger storage pool (presumably hepatic stellate cells) [R(15,16)]. Based on these 2 relative rates of input into plasma, 10% of the RBP combines on a 1:1 molar basis with retinol from compartment 14 and enters plasma RBP compartment 45 at the same rate as retinol entering plasma compartment 15 from hepatocyte compartment 14. The other portion of available apo-RBP (90%) is transferred from compartment 43 to stellate cells (compartment 44), where it can acquire retinol from stores (compartment 16) and enter plasma compartment 45 as holo-RBP, paralleling the rate of transfer of retinol from compartment 16 into compartment 15. In order to link the retinol tracer and tracee models and since apo-RBP was made rate limiting, we made the mobilization of retinol from hepatocytes into plasma [(L(5,4) for tracer, which was equal to that for tracee, L(15,14)] dependent on available apo-RBP. We did this by taking the assigned value for L(5,4) and making it a function of both the steady state mass of apo-RBP in compartment 43 and also the potentially changing value for that mass; thus, if the RBP input changed, so would L(5,4). That is, L(5,4) = [assigned L(5,4)/steady state M(43)] x F(43), the time-variant value for M(43). The same was done for retinol mobilization from hepatic stellate cell stores [L(15,16)]; thus, L(15,16) = [assigned L(15,16)/steady state M(44)] x F(44), where F(44) is the time-variant value for M(44) (see **Supplemental WinSAAM Deck 1**).

Then, we altered the normal steady state by imposing 4 different perturbations that reflect conditions of inflammation which might be observed in humans: chronic inflammation initiated at the time of tracer dosing or acute inflammation that occurred at the time of dosing or 3 or 9 days later. We assumed that since the synthesis of RBP decreases during inflammation, the levels of RBP in hepatocytes and stellate cells would decrease from their constitutive steady state (constant) levels and that after recovery from inflammation, there would be a condition-dependent return to the pre-inflammation RBP input rate; further, we assumed that changes in plasma retinol concentrations would follow similar but slightly delayed time courses. For chronic inflammation, we assigned an RBP input rate that was half the steady state value and made this reduction persist for the duration of the study. For acute inflammation initiated on day 0, and based on extrapolation from limited published observations (16), we modeled transient decreases in RBP input and plasma retinol concentrations, with both parameters returning to the normal steady state levels by day 7. For acute inflammation induced at the later times, we incorporated the same transient changes in RBP input and plasma retinol.

To produce the responses in the apo-RBP input rate described in the preceding paragraph, we used a multiexponential function (Equation 2 below) that would provide the robustness and flexibility to simulate responses to different forms of inflammation. This equation is similar to that developed by author MHG during work with von Reinersdorff et al. (27) to describe the observed effect of a large dose of ingested [^13^C]retinyl palmitate on plasma [^12^C]retinol; in Gieng et al. (14), a similar approach was used to mimic the response of plasma retinol to lipopolysaccharide-induced inflammation in rats. Here, to produce the desired responses in apo-RBP input [U(43)] (see Supplemental WinSAAM Deck 1), we specified that U(43) = P(43) x G(43), where P(43) = R(15,14) + R(15,16) (i.e., the rate of secretion of diet-derived retinol from hepatocytes plus recycling of retinol from stores) in the steady state and during acute inflammation and is half that value during chronic inflammation, and G(43) = 1 during chronic inflammation and is defined by Equation 2 during acute inflammation:

(2) $$\begin{eqnarray*}
{\rm G}\left( {{\rm 43}} \right) &=& {\rm P}\left( {{\rm 11}} \right) \times {\rm EXP}\left[ {{\rm - P}\left( {\rm 1} \right) \times {\rm T}} \right]{\rm + EXP}\left[ {{\rm{ - P}}\left( {\rm 2} \right) \times {\rm T}} \right]\nonumber\\
&& + \left( {{\rm P}\left( {{\rm{12}}} \right)- {\rm{\{ EXP}}\left[ {{\rm{ - P}}\left( {\rm 3} \right) \times {\rm T}} \right]{\rm + EXP}\left[ {{\rm - P}\left( {\rm 4} \right) \times {\rm { T}}} \right]{\rm{\} }}} \right)
\end{eqnarray*}$$

Here, EXP is the natural log e, T is time, and P(I) are adjustable parameters whose values were determined by fitting observed data for plasma retinol concentrations following vaccine-induced inflammation in Bangladeshi children (unpublished observations; SM Ahmad, International Centre for Diarrhoeal Disease Research, Dhaka, Bangladesh). From a biochemical perspective, Equation 2 describes how the rate constant for RBP synthesis is reduced for a period of time with the onset of acute inflammation but then rebounds to normal, whereas the rate constant for RBP degradation is unchanged. For acute inflammation induced at 3 or 9 days, T in Equation 2 had to be modified so that G(43) was initiated at those later times. To accomplish this, T was replaced with TH, a second independent variable that can be used in WinSAAM, with TH starting at day 3 or 9 (see **Supplemental WinSAAM Deck 2**).

After the retinol tracee and RBP models had been developed for subject 2, we applied the same models to the other 5 subjects.

### Calculation of TBS by RID in the steady state and under conditions of inflammation

To assess the impact of inflammation on RID-predicted TBS, we first used the steady state retinol tracer model for each subject to simulate time-variant values for both SA_p_ and the composite RID coefficient *FaS*, and we applied those values in RID Equation 1 to predict the time-invariant steady state value for TBS. Then, we compared these estimates to values predicted when inflammation was added as a perturbation of the normal condition, using the same steady state values for *FaS* (*FaS_ss_*) but with those for SA_p_ predicted by the model under the various conditions of inflammation. We used steady state values for *FaS*, since when RID is applied in the field, values for *FaS* are obtained from the literature (11) or from a super-subject model for control subjects assuming steady state (13). To validate our model, we also calculated RID-predicted TBS using time-dependent values of *FaS* determined during inflammation (rather than with *FaS_ss_*), along with the corresponding values of SA_p_, to confirm that these parameters would predict the correct time-invariant assigned values for TBS. Finally, we systematically investigated the impact of changes in the components of *FaS* and SA_p_ on TBS predictions to pinpoint the factor(s) that lead to incorrect predictions of TBS during inflammation.

### Data management

Data were managed in Microsoft Excel. Figure 1 was created in Microsoft PowerPoint and the image quality was improved using Adobe Photoshop; all other figures were created using GraphPad Prism 7.0 for Windows.

## Results

### Apo-RBP input, plasma retinol, and RID predictions of TBS without and with inflammation

**FIGURE 2:**
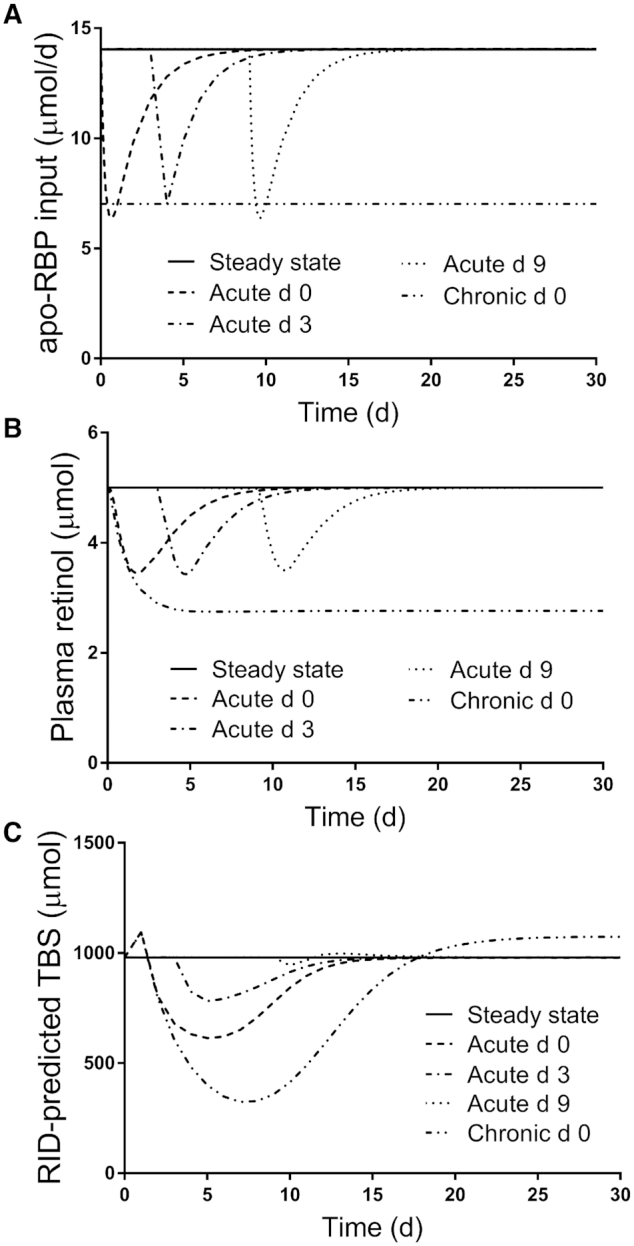
Simulated responses for (A) apo-RBP input rate, (B) plasma retinol pool size, and (C) RID-predicted TBS versus time for theoretical subject 2 in the steady state and during acute or chronic inflammation. Stable isotope-labeled retinol was ingested on day 0 and TBS was predicted using Equation 1, with the composite RID coefficient *FaS* calculated from the steady state tracer model. Inflammation was introduced into the steady state models as a perturbation at either time 0 (chronic and acute) or on day 3 or 9 (acute). The models are shown in Figure 1. Abbreviations: RBP, retinol-binding protein; RID, retinol isotope dilution; TBS, vitamin A total body stores.

**FIGURE 3:**
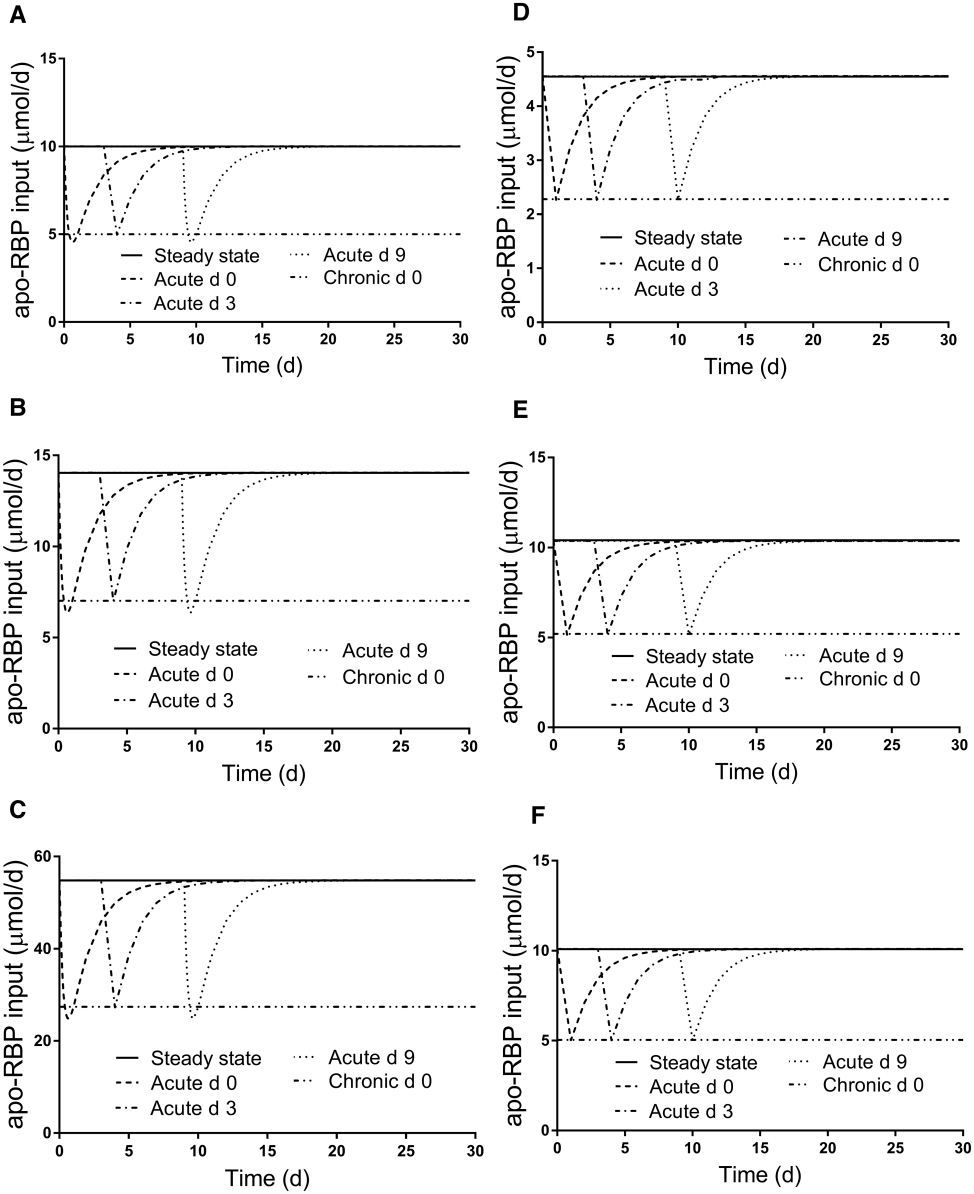
Simulated responses for apo-RBP input rate versus time for theoretical subjects (A) 1, (B) 2, (C) 3, (D) 4, (E) 5, and (F) 6 in the steady state and during acute or chronic inflammation. Stable isotope-labeled retinol was ingested on day 0 and inflammation was introduced into the steady state models as a perturbation at either time 0 (chronic and acute) or on day 3 or 9 (acute). The models are shown in Figure 1; assigned values for subject characteristics and kinetic parameters are presented in Supplemental Table 1. Abbreviation: RBP, retinol-binding protein.

**FIGURE 4:**
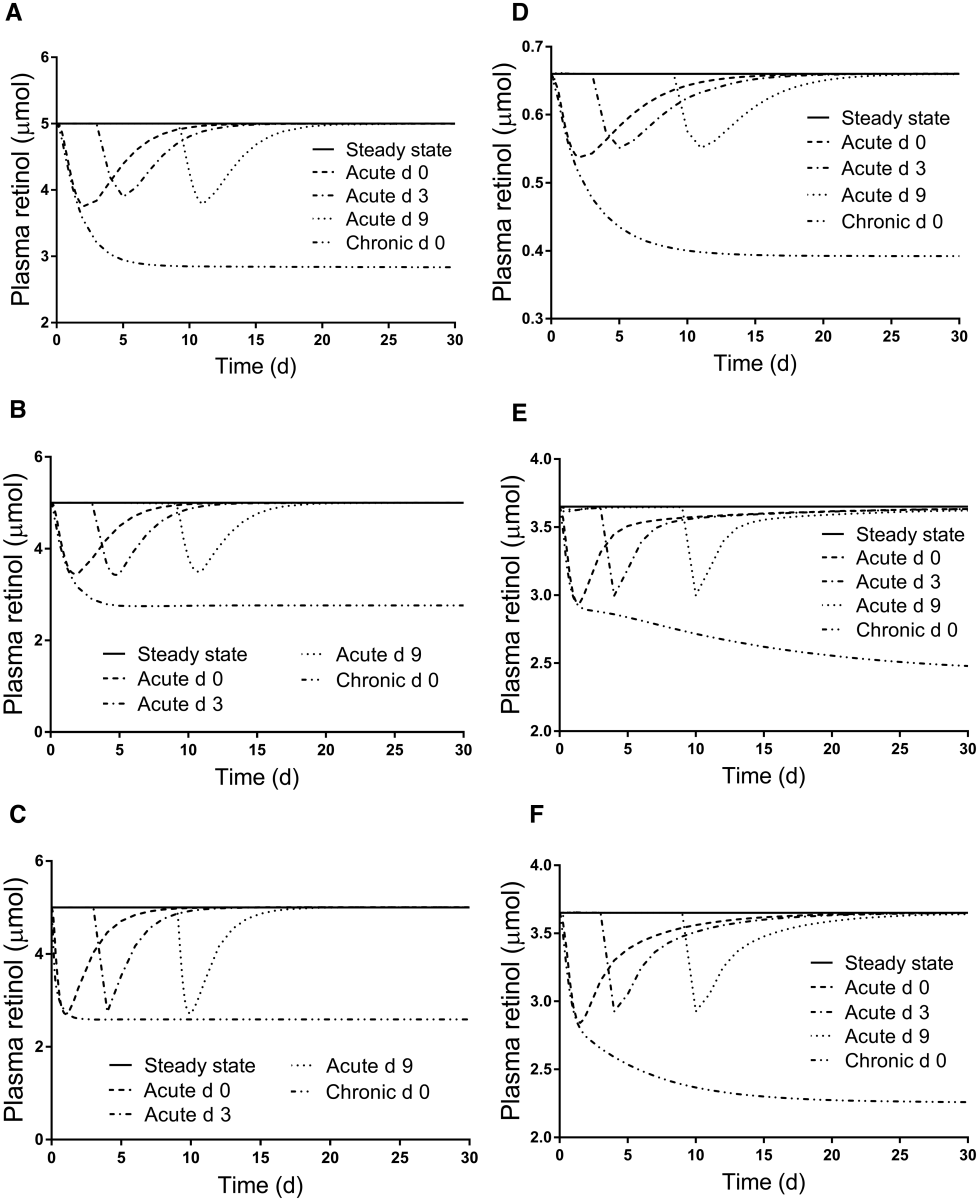
Simulated responses for plasma retinol pool size versus time for theoretical subjects (A) 1, (B) 2, (C) 3, (D) 4, (E) 5, and (F) 6 in the steady state and during acute or chronic inflammation. Stable isotope-labeled retinol was ingested on day 0 and inflammation was introduced into the steady state models as a perturbation at either time 0 (chronic and acute) or on day 3 or 9 (acute). The models are shown in Figure 1; assigned values for subject characteristics and kinetic parameters are presented in Supplemental Table 1.

**FIGURE 5:**
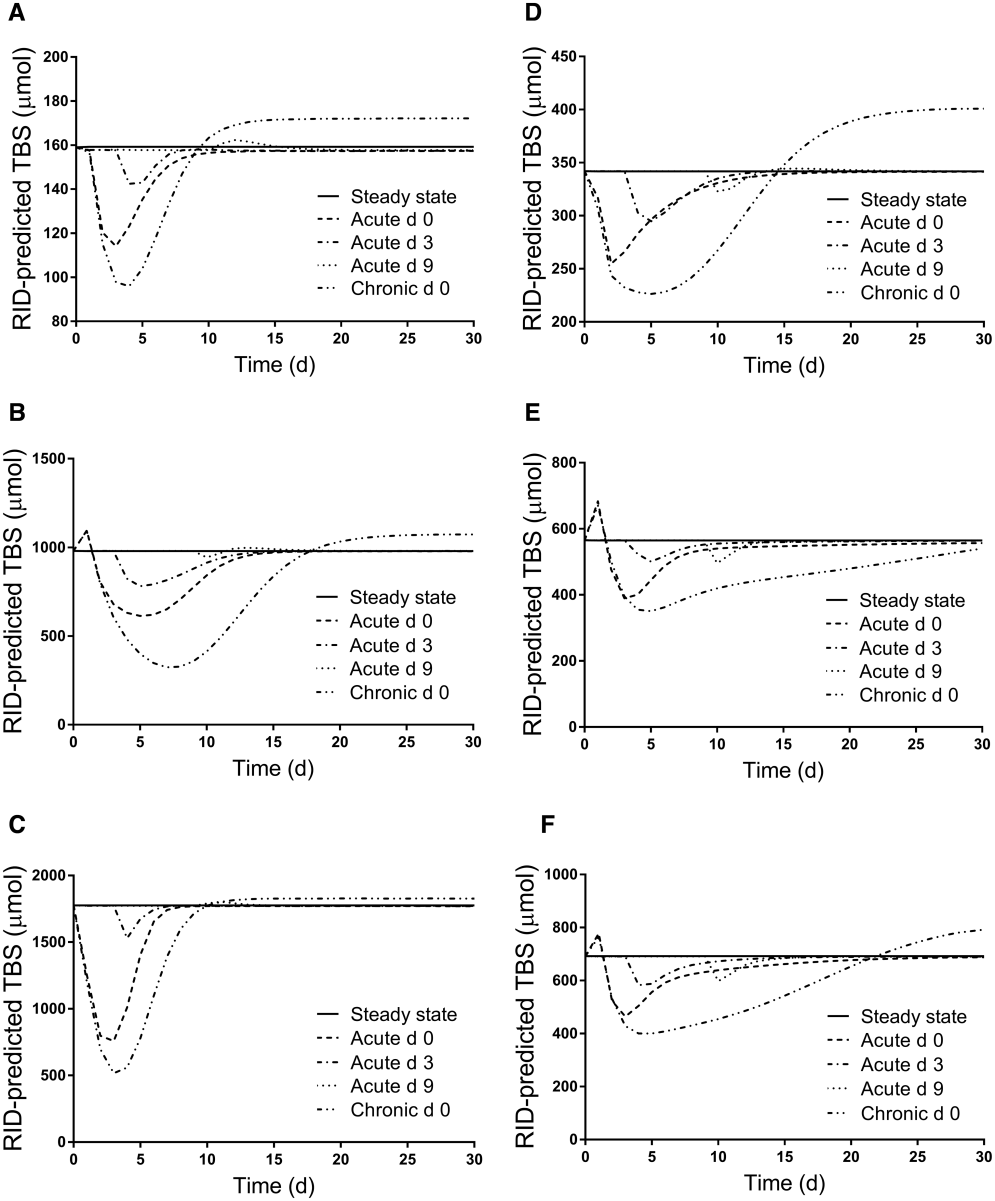
Simulated responses for RID-predicted TBS versus time for theoretical subjects (A) 1, (B) 2, (C) 3, (D) 4, (E) 5, and (F) 6 in the steady state and during acute or chronic inflammation. Stable isotope-labeled retinol was ingested on day 0 and inflammation was introduced into the steady state models as a perturbation at either time 0 (chronic and acute) or on day 3 or 9 (acute). The models are shown in Figure 1; assigned values for subject characteristics and kinetic parameters are presented in Supplemental Table 1. Abbreviations: RBP, retinol-binding protein; RID, retinol isotope dilution; TBS, vitamin A total body stores.

**Figure 2** shows model-predicted results versus time after tracer administration for RBP input rate, plasma retinol pool size, and RID-calculated TBS in the steady state, as well as in response to chronic and acute inflammation for subject 2; results for all subjects are presented in **Figures 3** –**5**. As indicated in Figure 2A for subject 2, under normal steady state conditions, the RBP input rate [(U(43); Figure 1B] was constant over the study at 14 μmol/d, as was plasma retinol pool size [M(5)] at the assigned value of 5 μmol; the curves for these parameters are similar because of the assumptions included in the model, including the approximation that retinol is in a 1:1 molar ratio with RBP in circulation. TBS calculated by RID was also constant at the model-predicted value of 980 μmol (Figure 2C). Note that subject-specific values for U(43) and M(5) were used for the other subjects.

As expected, initiation of inflammation affected all 3 parameters. The RBP input rate (Figure 2A for subject 2) was set at half its steady state value in the case of chronic inflammation initiated on day 0, and it remained at that level throughout the study. In contrast, following acute inflammation, the RBP input rate rapidly decreased to ∼50% of the steady state value after inflammation was initiated, whether initiation was on day 0, 3, or 9, and then it returned to the steady state value ∼1 week after the onset of inflammation. The plasma retinol pool size (Figure 2B) decreased rapidly in response to all 4 of the inflammation conditions tested, since our model specified apo-RBP–dependent secretion of retinol from hepatocytes and stellate cells. During chronic inflammation, plasma retinol declined to 55% of the steady state value by 10 days and then remained at that level. In contrast, but similar to the response in the apo-RBP input rate, plasma retinol fell to ∼70% of its steady state value when acute inflammation was initiated at 0, 3, or 9 days before returning to the steady state value ∼1.5 weeks after the onset of inflammation. Also, for acute inflammation induced on day 0, the fraction of plasma retinol input from “stellate cells” (set at 90% in the steady state) fell to 86% just after 2 days, returning to 90% by day 9.  Overall, it is important to emphasize that the impacts of acute inflammation on both apo-RBP input and plasma retinol pool size were similar whether the perturbation was started at the time of tracer administration (day 0) or on day 3 or 9. This is because both parameters were influenced by G(43), the equation used to control apo-RBP input for the 3 conditions of acute inflammation.

In the case of TBS calculated by RID (Figure 2C for subject 2), the effects of inflammation differed among the 4 conditions when calculated using *FaS_ss_* and the appropriate condition-dependent SA_p_. During chronic inflammation, TBS predictions (percentages above or below the steady state value) increased by 11% at day 1 and then decreased to 33% at 7 days before rising back to normal by day 18 and eventually reaching a plateau that was 10% above the steady state value. When acute inflammation was initiated on day 0, RID-predicted TBS increased by 12% at 1 day and then fell to 63% at 5 days before slowly returning to normal by day 19. When acute inflammation was initiated on day 3, RID-predicted TBS dropped to a low of 80% of the steady state value at day 5 before returning to normal by day 18. Finally, when acute inflammation was induced on day 9, the impact on TBS predicted by RID was greatly attenuated compared with other times, such that it was 3.7% lower on day 10, 102% at day 13, and normal again by day 21. Overall, and in contrast to the similar effect of acute inflammation induced on day 0 versus day 3 versus day 9 on apo-RBP input and plasma retinol, timing had a dramatic effect on RID-predicted TBS. Specifically, the depth of the nadir varied, with the greatest effect seen when inflammation was initiated at day 0; it was about half that when inflammation was started on day 3, and the difference was negligible for day 9. The time of the nadir (∼5 days) was similar whether inflammation was initiated at day 0 or 3. Importantly, regardless of the time of onset of acute inflammation, our models predict that if the RID test is applied 2 weeks or more after tracer administration, an accurate estimate of TBS should be obtained.

Figures 3 –5 show patterns for the 3 parameters in the steady state and under the 4 inflammation conditions for all subjects. For apo-RBP input (Figure 3), use of the function G(43) (Equation 2) resulted in similar patterns among subjects for each inflammation perturbation; for acute inflammation initiated at any of the 3 times, the nadir in response was at ∼50% of the steady state value. For plasma retinol pool size (Figure 4), chronic inflammation resulted in varied responses among subjects. In general, plasma retinol decreased and then increased to reach a new steady state value by 2 days to ∼2 weeks (although for subject 5, it was not until after 30 days). For acute inflammation, plasma retinol responses were similar across subjects because the same time-dependent G(43) function was used; however, the magnitude of the nadir varied, with reductions ranging from 20–45%. Finally, as shown in Figure 5, the response in RID-predicted TBS to inflammation was unique for each subject. However, in general across all subjects, chronic inflammation resulted in a decrease in RID-predicted TBS, reaching a nadir between 4 and 7 days, followed by a rebound above the steady state value; that is, there was a sustained overestimation of TBS, with the timing of recovery and magnitude of the later overestimation varying across individuals. In fact, for subject 5, predictions did not surpass the steady state value until after 35 days (data not shown). When acute inflammation was initiated at day 3 versus day 0, RID-predicted TBS showed smaller reductions for all subjects and, over all subjects, acute inflammation initiated at day 9 had a minimal effect on RID-calculated TBS.

### Which component of the RID equation is responsible for inflammation-related errors in prediction of TBS?

As described in Methods, the current analysis was done using *FaS_ss_* to predict TBS using RID Equation 1. The observed effects of inflammation on RID-predicted TBS led us to ask whether 1 or both components of the equation (SA_p_ or *FaS*) account for the changes from steady state TBS predictions. As detailed in (28), factor *Fa* (or fraction of the dose in stores at time *t*) appears twice in Equation 1: as *Fa* per se and also as a component of factor *S* whose denominator (SA_s_) = *Fa*/TBS. Thus, *Fa* cancels out of the relationship and anything that affects only *Fa* will not influence the composite coefficient *FaS*. Since it is not likely that vitamin A stores actually change much with inflammation over the short duration of an RID study, it is reasonable to conclude that inflammation-related differences in predicted TBS are due to effects on SA_p_. Note that if we use model-predicted values for *FaS* and SA_p_ that were calculated from the model during any of the conditions of inflammation described here, the correct value for TBS is predicted by Equation 1 at any time (data not shown).

To understand inflammation-related changes in SA_p_ over time, we need to examine changes in both the numerator [fraction of the dose in plasma; F(5)] and the denominator [plasma retinol pool size; M(5)]. In the case of acute inflammation initiated at the time of tracer dosing, and continuing to use subject 2 as an example, we found (data not shown) that at day 1, the ratio of F(5) during inflammation to that in the steady state was 0.68. This resulted from the reduced apo-RBP input that led to a slowing of tracer secretion from hepatocyte compartment 4 into plasma compartment 5; in contrast, the ratio for M(5) at day 1 of inflammation versus steady state was 0.76 due to reduced availability of apo-RBP for retinol secretion. That means the ratio for SA_p_ was 0.89 at day 1 and, since differences in SA_p_ and TBS are inversely related, the ratio for RID-calculated TBS would be 1.1. For M(5) (Figure 2B), the ratio (inflammation/steady state) reaches a nadir of 0.69 at day 2; when the TBS ratio is at a minimum on day 5, the M(5) ratio has increased to 0.90 and has further increased to 1 by day 11. With respect to F(5), as the delayed secretion of tracer from hepatocytes into plasma begins to catch up with and surpass the steady state secretion rate, the F(5) ratio becomes >1 (1.1) at day 3 and then increases to a peak at day 6 (ratio = 1.5) before slowly falling back to the steady state level by day 15. Combining these 2 responses and following SA_p_ [i.e., F(5)/M(5)] over time, the ratio reaches a maximum at day 5 (1.6), with the F(5) ratio at 1.4 and the M(5) ratio at 0.90. Of note, this is the time that RID-predicted TBS is at its lowest (63%) compared with the steady state value (Figure 2C).

## Discussion

In this work, we developed a compartmental model to link liver RBP input with hepatic secretion of RBP-bound retinol, and we used our model to evaluate the influence of inflammation, which is known to reduce hepatic RBP synthesis (15), on the prediction of vitamin A TBS by a standard RID method (i.e., using an individual subject's SA_p_ and a value for the coefficient *FaS* determined or assumed for a normal individual or group). To our knowledge, this is the first model that focuses on these processes, although model-based compartmental analysis has been previously used to study the effect of inflammation on vitamin A kinetics in rats (14, 16). In developing our model, we assumed that the balance point that maintains plasma retinol homeostasis depends on many factors, including the availability of apo-RBP in hepatocytes and liver stellate cells (29), and we incorporated current knowledge and assumptions about the effects of chronic or acute inflammation on apo-RBP input and plasma retinol concentrations (30). Because we used simulated data generated for theoretical subjects with known values for TBS, we were able to quantitate the effect of inflammation on RID-predicted TBS. This novel approach should be useful for studying the impact of various perturbations on vitamin A kinetics and metabolism in both animal models and humans, and it is likely applicable to other nutrients.

Our results confirm the validity of previously raised cautions related to applying the RID test in individuals with inflammation or when the presence or absence of infection has not been documented. We found that for acute inflammation, the magnitude of the effect on RID-predicted TBS depended on the timing and duration of the perturbation relative to the times of isotope administration and blood sampling for RID. Specifically, the largest effect on TBS predictions was observed when inflammation was initiated on the day of tracer dosing, and the later the onset of inflammation, the smaller the observed effect; in fact, the error in RID-predicted TBS when inflammation was initiated at 9 days was negligible. In contrast, for chronic inflammation induced on day 0, RID-predicted TBS was still ∼10% above the steady state value after 2 weeks, which may be an acceptable level of error. Overall, our results indicate that if a subject is free of inflammation based on plasma CRP and AGP measured at the time of tracer administration and if blood sampling for the RID test is done 2 weeks or more later, a reasonably accurate prediction of TBS is likely, independent of the inflammation status at the time of blood sampling for RID. This finding is likely attributable to the fact that once the tracer has mixed with endogenous vitamin A and enough time has elapsed for the tracer to have been mobilized from hepatocytes, the measured variable in the RID test (SA_p_ in Equation 1) will not be appreciably impacted; thus, RID-predicted TBS will be accurate. From a practical perspective, it is worth emphasizing that although applying the RID method later (i.e., obtaining the blood sample for measurement of SA_p_ 2 weeks or more after tracer administration) might require a larger dose of label (e.g., 2 mg) to ensure accurate analytical quantification, such a dose would delay the release of the tracer from hepatocytes but not perturb the overall system. As previously recommended (21), subjects should continue to consume a normal vitamin A intake after tracer administration. Another approach might be to investigate whether a “correction factor” could be established to adjust RID-predicted TBS obtained during inflammation to a more correct value, similar to the adjustments made to plasma RBP and retinol concentrations when biomarkers of inflammation such as CRP and AGP are detected (31). Finally, one could use the paired RID approach applied by Haskell et al. (32) to evaluate the impact of increased vitamin A intake on TBS to determine subjects’ TBS under control conditions and again after induction of inflammation.

For the model presented here, we included fixed values for U(43) in the steady state and in chronic inflammation; for conditions of acute inflammation, U(43) was set equal to the function G(43) (Equation 2) to cause temporal changes in apo-RBP input rate. We have previously used this type of equation to describe changes in vitamin A kinetics (14, 27, 33). Here, inclusion of G(43) allowed for reductions in the apo-RBP input rate during acute inflammation and resulted in concomitant decreases in plasma retinol levels of ∼20–45% below the steady state value. These results are consistent with previous reports indicating that both retinol and RBP concentrations can be reduced by as much as 40% in the presence of inflammation caused by trauma or infection (31). As shown here, the equation for G(43) can accommodate various responses that might be observed during inflammation. Specifically, with 2 exponentials that describe the fall to a nadir and 2 that describe the recovery, as well as an adjustable parameter that describes the recovery plateau, Equation 2 includes 9 parameters that can be adjusted to give the shape, nadir, recovery, and recovery plateau that might be observed during inflammation. Further, WinSAAM's provision of a second independent parameter for time (TH in Equation 2 for acute inflammation initiated at day 3 or 9) was essential for simulating responses when inflammation was initiated at these later times.

We applied our current approach to investigate several additional concerns related to effects of inflammation on the accuracy of RID for estimating vitamin A status. First is evidence that inflammation leads to reductions in vitamin A absorption efficiency (34, 35). If so, predictions of TBS by RID would be affected, since absorption efficiency influences both *Fa* and SA_p_ in RID Equation 1. To look at the potential effect of changes in absorption efficiency on RID predictions of TBS, we simulated our model for subject 2 assuming lower tracer absorption (50% versus 74%). Results showed that when the correct model-predicted values for *FaS* and SA_p_ were used (i.e., with both reflecting reduced absorption efficiency), Equation 1 accurately predicted the steady state value for TBS. However, if *FaS_ss_* was used (reflecting an absorption efficiency of 74%) along with the values for SA_p_ obtained assuming reduced absorption, TBS was overestimated by 50%. Until more information is available on the impact of inflammation on vitamin A absorption, investigators should test for inflammation by measuring plasma CRP and AGP before subjects are given the tracer dose in an RID study; tracer should not be administered to those who test positive for inflammation until after recovery.

We also used our current method to ask how established (ongoing) chronic inflammation, as seen in rheumatoid arthritis, ulcerative colitis, Crohn's disease, obesity (36), and kwashiorkor (37), would affect RID-predicted TBS. When we simulated the model for subject 2 under the condition of chronic inflammation, it took 7 days to establish a new, lower steady state that reflected the reduction in apo-RBP input and the subsequent fall in plasma retinol concentrations; then we simulated administration of labeled retinol on day 7. Values for RID-predicted TBS (**Supplemental Figure 1**) were very similar to those seen when chronic inflammation was initiated at the time of tracer dosing, indicating that if the blood sample for measuring SA_p_ is obtained 2 weeks or more after tracer administration, a reasonably accurate estimate of TBS (likely within 20% of the real value) should be predicted by Equation 1.

In conclusion, we present here an expanded model for vitamin A metabolism that links retinol secretion from the liver to hepatic RBP synthesis and secretion; this model facilitated study of the influence of inflammation on prediction of vitamin A TBS by RID. The capacity of model-based compartmental analysis to describe and quantitate the effects of perturbations on vitamin A tracer and tracee kinetics suggests that this unique and powerful method may be useful in advancing understanding of the effects of alterations to normal metabolism in other systems of interest.

## Notes

Support for this work was provided by the Bill & Melinda Gates Foundation (project number OPP1115464).

Author disclosures: MHG, JLF, and JBG, no conflicts of interest.

Supplemental Table 1, Supplemental Figure 1, Supplemental WinSAAM Decks 1 and 2, and Supplemental References are available from the “Supplementary data” link in the online posting of the article and from the same link in the online table of contents at http://academic.oup.com/jn.

Present address for JLF: Division of Pharmacotherapy and Experimental Therapeutics, UNC Eshelman School of Pharmacy, University of North Carolina, Chapel Hill, NC 27599.

Abbreviations used: AGP, a1-acid glycoprotein; CRP, C-reactive protein; RBP, retinol-binding protein; RID, retinol isotope dilution; SA_p_, retinol specific activity in plasma; SA_s_, retinol specific activity in stores; TBS, vitamin A total body stores; WinSAAM, Windows version of Simulation, Analysis and Modeling software.
